# Exosomal miRNAs and lncRNAs: The Modulator Keys of Cancer-Associated Fibroblasts in the Genesis and Progression of Malignant Neoplasms

**DOI:** 10.3389/fcell.2021.717478

**Published:** 2021-11-29

**Authors:** Julio César Villegas-Pineda, Mélida del Rosario Lizarazo-Taborda, Adrián Ramírez-de-Arellano, Ana Laura Pereira-Suárez

**Affiliations:** ^1^ Doctorado en Ciencias Biomédicas, Departamento de Fisiología, Centro Universitario de Ciencias de la Salud, Universidad de Guadalajara, Guadalajara, Mexico; ^2^ Instituto de Investigación en Ciencias Biomédicas, Centro Universitario de Ciencias de la Salud, Universidad de Guadalajara, Guadalajara, Mexico; ^3^ Programa de Bacteriología y Laboratorio Clínico, Facultad de Ciencias de la Salud, Universidad de Santander, Cúcuta, Colombia; ^4^ Departamento de Microbiología y Patología, Centro Universitario de Ciencias de la Salud, Universidad de Guadalajara, Guadalajara, Mexico

**Keywords:** cancer, cancer-associated fibroblast, tumor microenvironment, exosomes, miRNAs, lncRNAs

## Abstract

The tumor microenvironment is made up of a universe of molecular and cellular components that promote or inhibit the development of neoplasms. Among the molecular elements are cytokines, metalloproteinases, proteins, mitochondrial DNA, and nucleic acids, within which the ncRNAs: miRNAs and lncRNAs stand out due to their direct modulating effects on the genesis and progression of various cancers. Regarding cellular elements, the solid tumor microenvironment is made up of tumor cells, healthy adjacent epithelial cells, immune system cells, endothelial cells, and stromal cells, such as cancer-associated fibroblasts, which are capable of generating a modulating communication network with the other components of the tumor microenvironment through, among other mechanisms, the secretion of exosomal vesicles loaded with miRNAs and lncRNAs. These ncRNAs are key pieces in developing neoplasms since they have diverse effects on cancer cells and healthy cells, favoring or negatively regulating protumoral cellular events, such as migration, invasion, proliferation, metastasis, epithelial-mesenchymal transition, and resistance to treatment. Due to the growing number of relevant evidence in recent years, this work focused on reviewing, analyzing, highlighting, and showing the current state of research on exosomal ncRNAs derived from cancer-associated fibroblasts and their effects on different neoplasms. A future perspective on using these ncRNAs as real therapeutic tools in the treatment of cancer patients is also proposed.

## Introduction

**FIGURE 1:**
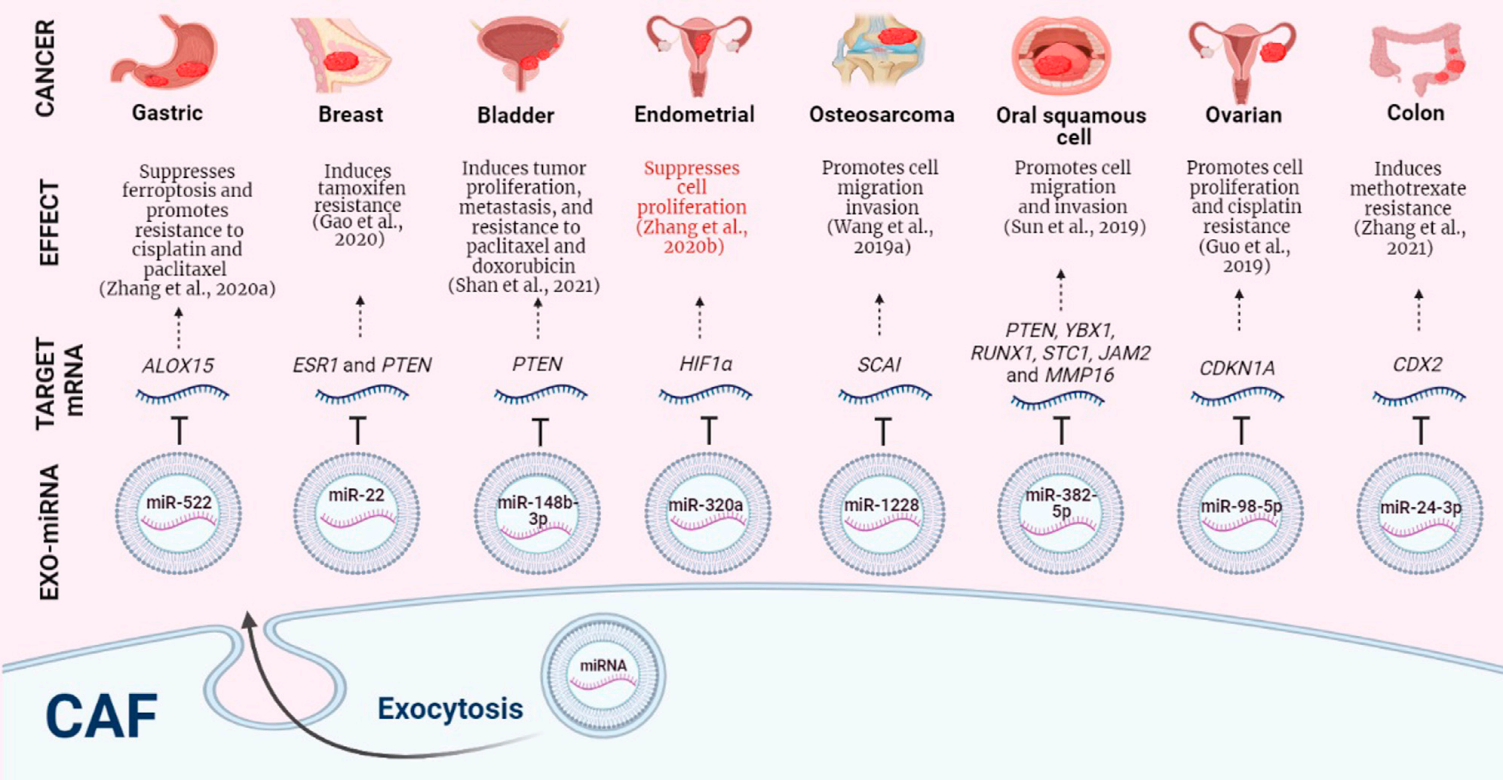
CAFs-derived exosomal miRNAs, their target mRNAs and effects on different neoplasms. Protumoral events in black, antitumor events in red. CAF: Cancer-associated fibroblast. EXO-miRNA: exosomal miRNA. Image created in BioRender.com.

Malignant tumors consist of cancer cells and tumor-associated host cells (De Wever et al., 2014), the interaction between tumor microenvironment (TME) and tumor cells plays a key role in cancer progression (Dayan et al., 2012; Ali et al., 2015; Shah et al., 2015; Vered et al., 2015; Ringuette Goulet et al., 2018; Zhang Y.-F. et al., 2019; Dou et al., 2020; Wu et al., 2020). Remarkably, the dynamic interplay between cancer-associated fibroblasts (CAFs), cancer cells, and healthy cells has an essential role during tumor initiation and growth (Zhao et al., 2016; Li K. et al., 2020; Lv et al., 2020). In recent years, exosomes have gained relevance due to their regulatory role in the carcinogenesis of different neoplasms (Nilsson et al., 2009; Ramteke et al., 2015; Donnarumma et al., 2017; Seo et al., 2018; Zhang Y. et al., 2019; Lee et al., 2020). Exosomes are MHC class I- and class II-bearing nanovesicles of endocytic origin; their size is in the range of 30–100 nm (Admyre et al., 2007; Nilsson et al., 2009). Exosomes can participate in intercellular communication between cells that make up the TME by transmitting intracellular cargoes (Achreja et al., 2017; Qin et al., 2019), their content can be miscellaneous and include macromolecules such as cytokines (Mashouri et al., 2019), metalloproteinases (Shimoda et al., 2014), proteins (Mathivanan et al., 2010; Zhang Y.-F. et al., 2019; Zhao et al., 2020), mitochondrial DNA (mtDNA) (Sansone et al., 2017) and can be enriched with different types of RNAs, such as messenger RNAs (mRNAs) (Gener Lahav et al., 2019), microRNAs [miRNAs or miR-, single-stranded non-coding RNAs (ncRNAs) of 20 nucleotides in length that are endogenously expressed (Beermann et al., 2016)] (Théry, 2011; Nouraee et al., 2016; Qin et al., 2019), about this type of ncRNAs, Dragomir et al., in their work entitled “*SnapShot: Unconventional miRNA Functions*,*”* mention that miRNAs can regulate gene expression at the post-transcriptional level in a conventional way by binding to mRNAs, resulting in the disintegration of target mRNAs and inhibition of translation, additionally, the authors also highlight in an exceptional way that through various mechanisms, such as the activation of Toll-like receptors, the upregulation of protein expression, the targeting of mitochondrial transcripts, the direct activation of transcription, among others, miRNAs can carry out their regulatory functions in an unconventional way (Dragomir et al., 2018), and long non-coding RNAs (lncRNAs, transcripts that are longer than 200 nucleotides and do not harbor protein-coding signatures (Beermann et al., 2016)) (Zhou et al., 2021). Exosomes can be produced by immune system cells (Elashiry et al., 2021), epithelial cells (Han et al., 2017), tumor cells (Gu et al., 2012; Cheng et al., 2017; Ding et al., 2018; Chen et al., 2019; Hu and Hu, 2019; Amit et al., 2020; Jung et al., 2020; Yang et al., 2020) and CAFs (Herrera et al., 2018; Principe et al., 2018; Liu et al., 2020). CAFs are resident stromal cells of the TME; they have a promoting effect on carcinogenesis and progression of different neoplasms (Luga and Wrana, 2013; Huang et al., 2019; Sun and Fu, 2019) by transferring exosomes carrying ncRNAs to cells of the TME (Richards et al., 2017; Fiori et al., 2019) (Figure 1).

For everything mentioned above, the present work is based on the recent findings on the protumoral and antitumoral effects of CAFs-derived exosomal miRNAs and lncRNAs; it focuses on analyzing and highlighting the importance of these ncRNAs in the genesis and progression of different cancers. At the same time, it shows the current situation of this new growing research area, and a future perspective on the use of these ncRNAs as real therapeutic tools in the treatment of cancer patients is also proposed.

## Exosomes: Inducing Factor in the Formation of CAFs and Tumor Microenvironment Modulator

Exosomes are among the most recognized inducers for generating CAFs (Li K. et al., 2020). CAFs are known as cells capable of secreting exosomes (You et al., 2019), and in turn, exosomes derived from cancer cells containing TGF-β can induce the generation of CAFs from stromal cells (Goulet et al., 2019). It has also been observed that exosomes derived from CAFs containing TGF-β activate the SMAD signaling pathway in cancer cells through a particular type of epithelial-mesenchymal transition (EMT), which increases their malignant behavior (Li et al., 2017), thus generating a loop between CAFs and exosomes. It has been shown that chronic lymphocytic leukemia cells-derived exosomes can transfer miR-146a to bone marrow-derived mesenchymal stem cells to generate CAFs; this is done by promoting EMT by targeting ubiquitin specific peptidase 16 (*USP16*) (Yang et al., 2020). Another cellular transition process in which exosomes are involved to originate CAFs is the endothelial-mesenchymal transition; this event was demonstrated by observing that melanoma-derived exosomes loaded with TGF-β induced the transition from human umbilical vein endothelial cells (HUVECs) to differentiated CAFs (Yeon et al., 2018). For their part, Baroni et al. demonstrated that triple-negative breast CAFs-derived exosomal miR-9 could generate a cell type with CAF-like properties in human breast fibroblasts, in which this miRNA enhanced the capacity for migration and invasion, as in breast cancer cell lines. Additionally, overexpression of this ncRNA in normal fibroblasts was able to promote tumor growth in a murine orthotopic xenograft model (Baroni et al., 2016).

It has also been observed that the exosomes produced by CAFs can modulate TME. For example, miR-21, which is packed in CAF-derived exosomes, induces the generation of monocytic myeloid-derived suppressor cells by activating STAT3 (Zhao et al., 2021). On a breast cancer model, exosomes produced by CAFs can also interfere with immunologic processes. In breast cancer cells treated with exosomes derived from CAFs, an increase of miR-92 was observed, which was essential for migration and invasion and correlated with the suppression of the immune cell function and the promotion of PD-L1 expression in these cells (Dou et al., 2020). Also, in the metastatic lung niche, a higher capacity to induce the transformation fibroblast into CAFs has been observed due to exosomal transport of miR-1247-3p from high-metastatic hepatocellular carcinoma cells; leading to a pro-inflammatory microenvironment promoted by CAFs, which secret IL-6 and IL8, among other cytokines (Fang et al., 2018).

Considering this evidence, the important reciprocal relationship between exosomes and CAFs in the latter’s self-generation and consequently in neoplasms’ development is manifest. This relationship could be explored in greater depth and considered a previous or potentiating stage of carcinogenesis to inhibit the exosomal release and block exosomal molecules essential for the initiation, establishment, and progression of different cancers.

## Cellular Effects of CAFs-DERIVED Exosomal miRNAs and lncRNAs on Key Events for Genesis and Progression of Neoplasms

### Migration and Invasion, Processes Upregulated by CAFs-Derived Exosomal ncRNAs

Migration and invasion of cancer cells are essential for the establishment of the malignant neoplasm. Sun et al. isolated CAFs from patients with oral squamous cell carcinoma (OSCC) and showed that in tongue squamous cell carcinoma CAL-27 cells, CAFs could promote these two events through the transfer of exosomal miR-382-5p, which facilitated the OSCC progression. Despite not having biologically determined the interaction, an analysis *in silico* predicted *PTEN*, *YBX1*, *RUNX1*, *STC1*, *JAM2*, and *MMP16* as candidate target genes for this miRNA (Sun et al., 2019). Another exosomal miRNA that has been shown to promote cell migration and invasion is miR-1228; this small non-coding regulatory RNA was enriched in exosomes secreted by CAFs and could downregulate endogenous *SCAI* mRNA and protein level in osteosarcoma, contributing to carcinogenesis of this neoplasm (Wang J.-W. et al., 2019).

CAFs-derived exosomes can also carry lncRNAs; LINC00659 is an example of this; it was found enriched in CAFs-derived exosomes and was shown to have multiple effects on human colorectal cancer (CRC) cells, it was able to induce cell proliferation, migration, invasion, and EMT *in vitro*. Furthermore, it was determined that these events were promoted by directly interacting with the tumor suppressor miR-342-3p to increase ANXA2 expression in CRC cells (Zhou et al., 2021).

The protumoral effects exerted by various CAFs-derived exosomal miRNAs and lncRNAs enhance the migration and invasion of cancer cells and convert them into key pieces within the universe of participants in developing neoplasms.

### CAFs-Derived Exosomal ncRNAs Promote Proliferation and Metastasis, Essential Malignant Characteristics for Tumor Progression

Among the multiple protumoral effects that CAFs-derived exosomal ncRNAs exert on cancer cells are the promotion of cell proliferation and metastasis. Chen et al. determined that CAFs isolated from samples of patients with invasive ductal carcinoma were capable of transferring exosomal miR-500a-5p to breast cancer cell lines, this miRNA bound to ubiquitin-specific peptidase 28 (*USP28*) promoting cell proliferation and metastasis in a nude mouse xenograft model (Chen et al., 2021). It has also been shown that miR-181d-5p can promote various protumoral cellular events in breast cancer cells, such as proliferation, invasion, and migration. If not enough, it can also induce EMT, antagonize apoptosis *in vitro* of breast cancer cells and promote tumor growth in nude mice xenografted with MCF-7 cells *via* downregulation of the transcription factors CDX2 and HOXA5 (Wang et al., 2020).

Another important effect carried out by CAFs exosomal ncRNAs is the reprogramming of metabolic pathways. Although to date, this aspect has not yet been studied in-depth, there is a report where a protumoral role is proposed for lncRNA SNHG3, which positively regulated pyruvate kinase isozymes M1/M2 (PKM) expression, inhibited mitochondrial oxidative phosphorylation, increased glycolysis, and proliferation of breast tumor cells through a mechanism similar to a molecular sponge for antitumoral miR-330-5p (Li Y. et al., 2020); this highlights the multifunctionality that CAFs-derived exosomal ncRNAs can exert on various processes related to carcinogenesis and tumor progression.

Considering the evidence reported, CAFs-derived exosomal miRNAs and lncRNAs can participate directly in tumor growth and the dissemination of cancer cells to other anatomical sites adjacent or far from the primary site, which could cause functional compromise of multiple organs and consequent serious complications in cancer patients.

### Treatment Resistance Generated by CAFs-Derived Exosomal miRNAs and lncRNAs

CAFs are an important factor in generating tumor resistance to treatment (Fiori et al., 2019); this protumoral feature is associated with worsening cancer patients’ prognosis (Domvri et al., 2020; Huang et al., 2020). CAFs possess chemoresistance by innate nature and, it has been observed that in the presence of gemcitabine, a cytotoxic anticancer chemotherapy drug, they can transfer this characteristic to cancer cells. CAFs exposed to this antineoplastic drug could increase the release of miR-146a-loaded exosomes; in *in vitro* assays, this miRNA transmitted gemcitabine resistance to pancreatic cancer epithelial cell lines, promoting cell proliferation and survival. In the same work, it was shown that miR-146a is directly regulated by the promoter binding transcription factor, Snail, which acts as a chemoresistance-inducing factor, and that it was also found to be overexpressed in human pancreatic CAFs-derived exosomes (Richards et al., 2017). Recently, Gao et al. identified and characterized a subtype of CAFs which particularly has the presence of the cell surface protein CD63; these CD63^+^ CAFs are capable of secreting exosomes enriched in miR-22, which can bind to their targets, *ER* α and *PTEN*, and deregulate their expression, thus conferring tamoxifen resistance to breast cancer cells (Gao et al., 2020).

Gemcitabine and tamoxifen are not the only drugs for which CAFs-derived exosomal miRNAs generate resistance in cancer cells; in fact, a considerable number of reports show that the cytotoxic effect caused by cisplatin in cancer cells can be diminished or eliminated by the action of CAFs-derived exosomal ncRNAs. It has been shown that, in cisplatin-sensitive ovarian cancer cells, CAFs-derived exosomal miR-98-5p was capable of binding to cyclin-dependent kinase inhibitor 1A (*CDKN1A*) to inhibit its expression, which increased ovarian cancer cell proliferation and cell cycle entry, suppressed cell apoptosis, and promoted cisplatin resistance *in vitro* and in a xenotransplanted nude mouse model (Guo et al., 2019). The intrinsic resistance that CAFs have towards cisplatin also favors the progression of head and neck cancer (HNC). It has been observed that CAFs can transfer exosomal miR-196a to HNC cells, which generated cell survival, proliferation, and inhibition of apoptosis, also conferred cisplatin resistance by targeting *CDKN1B* and *ING5*, cell cycle inhibitor and tumor suppressor molecules, respectively. Additionally, high levels of exosomal miR-196a in plasma were clinically correlated with poor overall survival and chemoresistance in patients with HNC (Qin et al., 2019).

There is a great diversity between the protumoral effects generated by CAFs-derived exosomal miRNAs and lncRNAs; it has been shown that miR-522 is capable of inhibiting ferroptosis, a novel mode of non-apoptotic cell death induced by a build-up of toxic lipid peroxides (lipid-ROS) in an iron-dependent manner, in gastric cancer cells by targeting *ALOX15* and blocking lipid-ROS accumulation. Additionally, assays performed in an orthotopic implantation model in nude mice to evaluate gastric tumor growth and chemosensitivity led the authors to suggest that CAFs-derived exosomes containing miR-522 promote a new mechanism of acquired chemoresistance to cisplatin through an intercellular pathway, comprising USP7, hnRNPA1, miR-522, and ALOX15 (Zhang H. et al., 2020).

On the other hand, it has been observed that miR-423-5p-loaded exosomes derived from CAFs can decrease the chemosensitivity of prostate cancer cells and increase the resistance of cells resistant to taxanes; in addition, it was shown that the inhibition of this miRNA enhanced the drug sensitivity of prostate cancer cells in a tumor xenograft model in nude mice. These protumoral events were favored due to the inhibitory effect of miR-423-5p on *GREM2* and the impact exerted on the TGF-β pathway (Shan et al., 2020). In another type of neoplasm, in bladder cancer, it was found that miR-148b-3p can also induce resistance to treatment. Shan et al. validated *PTEN* as a target of miR-148b-3p; the negative dysregulation of *PTEN* promoted metastasis, EMT, and resistance to doxorubicin and paclitaxel both in *in vitro* assays using bladder cancer cells and in *in vivo* assays using a xenograft mouse model. These events were generated due to the tumor-promoting effects of miR-148b-3p *via* the Wnt/β-catenin pathway (Shan et al., 2021).

CAFs-derived exosomal miRNAs have also shown an inhibitory effect on the cytotoxic action that methotrexate exerts on the metabolism of colon cancer cells. In an *in vivo* model of colon cancer, it was shown that miR-24-3p induced resistance to this drug, favoring tumor growth under treatment of methotrexate by down-regulating the CDX2/HEPH axis (Zhang et al., 2021).

As mentioned previously, CAFs are also capable of secreting exosomal lncRNAs that promote tumor progression. Such is the case of exosomal lncUCA1, which conferred resistance to cisplatin in vulvar squamous cell carcinoma cells. Mechanistically, lncUCA1 functioned as a sponge for miR-103a, a miRNA with antitumoral function in various human cancers, this sequestration of miR-103a by lncUCA1 promoted the expression of WEE1 G2 checkpoint kinase (*WEE1*), a direct target of miR-103a, enhancing tumor growth and cisplatin resistance in a BALB/c nude xenograft model (Gao et al., 2021). Another CAFs-derived exosomal lncRNA with protumoral effect is H19, highly expressed in tumors generated in an azoxymethane (AOM)/dextran sodium sulfate (DSS) model of colitis-associated cancer, as well as in CRC samples from patients in different tumor-node-metastasis stages. Additionally, this lncRNA promoted the stemness of CRC stem cells, increased the frequency of tumor-initiating cells, and promoted the resistance of CRC cells to oxaliplatin both *in vitro* and *in vivo*. Mechanistically, H19 activated the β-catenin pathway by acting as an endogenous competitor for miR-141, a miRNA with antitumoral effect, in CRC cells (Ren et al., 2018).

These findings propose miRNAs and lncRNAs as important obstacles to achieving a successful chemotherapeutic treatment; subject to further studies confirming this evidence, the elimination or reduction of CAFs-derived exosomal ncRNAs that generate chemoresistance should be considered as a new oncological therapeutic strategy.

### Exosomal miRNAs With Antitumoral Effects

miRNAs have generally been reported as protumoral molecules; however, some reports suggest that their silencing or elimination could favor tumor development. A feature shared between the antitumoral miRNAs is that they are significantly reduced in CAFs-derived exosomes. *In vitro* and *in vivo* studies carried out by Li et al. revealed that miR-148b could function as a tumor suppressor, the downregulation of this miRNA induced EMT, migration, invasion, and increased MMP-9 activity in endometrial cancer cell lines, as well as metastasis in a nude mouse model, additionally, DNA methyltransferase 1 (*DNMT1*) was determined as the target gene of miR-148b (Li et al., 2019). This enzyme has also been associated with tumorigenesis in breast cancer due to its indispensable role in the maintenance of mammary stem/progenitor cell and cancer stem cell maintenance (Pathania et al., 2015), and chemoresistance to cisplatin in human non-small cell lung cancer cell lines (Sui et al., 2015). Another study also carried out in endometrial cancer showed that the CAFs-derived exosomal miR-320a inhibited the proliferation of cell lines by direct targeting and downregulation of hypoxia-inducible factor 1-α (*HIFα*) (Zhang N. et al., 2020). This transcription factor has been strongly related to metastasis, angiogenesis, poor patient prognosis, and tumor resistance therapy (Masoud and Li, 2015). In another neoplasm typical of women, the CAFs-derived exosomal miR-4516 was isolated from tumor tissue obtained from a patient with invasive breast ductal carcinoma; this miRNA suppressed the proliferation of breast cancer cell lines by targeting *FOSL1* (Kim et al., 2020).

The antitumoral effects that some miRNAs possess have not only been reported in gynecological cancers. *In vitro* and *in vivo* studies carried out in human hepatocellular carcinoma cell lines and in a nude mouse model, respectively, showed that miR-320a could function as an antitumoral miRNA, whose binding to *PBX3* affected the protein expression of cyclin-dependent kinase 2 (CDK2) and MMP-2 due to the reduction of the phosphorylation of ERK1/2. In this way, the miR-320a/PBX3 axis suppressed essential events for cancer progression such as EMT, cell proliferation, migration, invasion, tumorigenesis, and metastasis (Zhang et al., 2017). miR-3188 is another miRNA that has an antitumoral effect; its loss of CAFs-derived exosomes contributed to the malignancy of HNC cells, increasing cell proliferation, migration, and invasion, and inhibited apoptosis by derepressing its target B-cell lymphoma 2 (*BCL2*) mRNA, this miRNA was also able to inhibit tumor growth in a BALB/c nude mice model (Wang X. et al., 2019).

Another of the few reports in which evidence is presented about the antitumoral effect of miRNAs is made by Li et al. It was observed that the overexpression of CAFs-derived exosomal miR-34a-5p suppressed the tumorigenesis of OSCC cells in an immunodeficient BALB/c mice subcutaneous tumor model. Additionally, in OSCC cell lines, it was shown that this miRNA is capable of binding directly to *AXL,* thus modulating the AKT/GSK-3β/β-catenin signaling pathway; this event reduced proliferation, migration, and invasion, and decreased nuclear translocation of β-catenin, which led to decreased expression of Snail, a transcription factor of MMP-2 and MMP-9, important for EMT (Li et al., 2018).

Considering the reported evidence of some miRNAs’ antitumoral effects in gynecological and non-gynecological cancers, these ncRNAs could become valuable tools to inhibit essential points in carcinogenesis and disease progression.

## CAFs-DERIVED Exosomal miRNAs and lncRNAs as Candidates for Therapeutic Tools and Biomarkers

Given the importance of protumoral and antitumoral miRNAs and lncRNAs in processes such as cell proliferation, migration, invasion, EMT, metastasis, metabolism, resistance to treatment (Table 1), these exosomal ncRNAs derived from CAFs have been suggested as strong candidates that could be used as new targets or therapeutic tools in breast, ovarian, vulvar squamous cell, endometrial, head and neck, pancreas, oral squamous cell, gastric, bladder, colon, liver, prostate cancer, as well as biomarkers of clinical relevance; CAFs-derived exosomal ncRNAs can be considered cornerstones in neoplastic processes.

**TABLE 1:** Cellular and tumor effects and mechanisms exerted by exosomal miRNAs and lncRNAs derived from CAFs in various cancers.

Exosomal ncRNAs Tumor effect	Cellular effects	Mechanisms	Cancer	References
miR-92	Promotes apoptosis and impairs proliferation of T cells, increases proliferation and migration of breast cancer cells, and facilitates tumor progression	Targets *LATS2* and modulates the LATS2-YAP1 axis generating an incremented expression of PD-L1	Breast	Dou et al. (2020)
PRO-TUMOR				
miR-196a	Promotes cell proliferation and confers cisplatin resistance	Targets and downregulates *CDKN1B* and *ING5*	Head and neck	Qin et al. (2019)
PRO-TUMOR				
lncRNA LINC00659	Promotes cell proliferation, migration, invasion, and EMT progression	Interacts directly with miR-342-3p to increase ANXA2 expression	Colorectal	Zhou et al. (2021)
PRO-TUMOR				
lncRNA-CAF	Increases cell proliferation and promotes tumor growth	Stabilizes and up-regulates cytokine IL-33 to reprogram CAFs	Oral squamous cell	Ding et al. (2018)
PRO-TUMOR				
miR-500a-5p	Enhances cell proliferation and induces metastasis	Binds to tumor suppressor ubiquitin-specific peptidase 28 (*USP28*)	Breast	Chen et al. (2021)
PRO-TUMOR				
miR-181d-5p	Induces cell proliferation, invasion, migration, and EMT, antagonizes apoptosis *in vitro,* and promotes tumor growth *in vivo*	Downregulates the expression of the transcription factors CDX2 and HOXA5	Breast	Wang et al. (2020)
PRO-TUMOR				
miR-423-5p	Promotes chemotherapy resistance	Targets *GREM2* to inhibit its expression and favors the TGF-β pathway	Prostate	Shan et al. (2020)
PRO-TUMOR				
lncRNA UCA1	Enhances tumor growth and chemoresistance	Favors the expression of WEE1 through sponging miR-103a	Vulvar squamous cell	Gao et al. (2021)
PRO-TUMOR				
lncRNA H19	Promotes stemness and chemoresistance	Activates the β-catenin pathway, acting as a competing endogenous RNA sponge for miR-141	Colorectal	Ren et al. (2018)
PRO-TUMOR				
lncRNA SNHG3	Inhibits mitochondrial oxidative phosphorylation, increases glycolysis, and enhances cell proliferation	Functions as a molecular sponge of miR-330-5p to regulate the expression of PKM	Breast	Li et al. (2020b)
PRO-TUMOR				
miR‐148b	Inhibits EMT and reduces cell invasion and metastasis	Directly binds to *DNMT1* and decreases MMP-9 activity	Endometrial	Li et al. (2019)
ANTI-TUMOR				
miR-4516	Suppresses cell proliferation	Targets *FOSL1,* proliferation-related gene	Breast	Kim et al. (2020)
ANTI-TUMOR				
miR-320a	Inhibits EMT, cell proliferation, migration, invasion, tumorigenesis, and metastasis	Binds to *PBX3*, suppresses the activation of the MAPK pathway, affects the expression of CDK2, and MMP2 proteins due to reduced phosphorylation of ERK1/2	Hepatocellular	Zhang et al. (2017)
ANTI-TUMOR				
miR-3188	Reduces cell proliferation, promotes apoptosis, and inhibits tumor growth	Directly targets *BCL2*	Head and neck	Wang et al. (2019b)
ANTI-TUMOR				
miR-34a-5p	Inhibits cell proliferation, migration, invasion, EMT, and tumorigenesis	Targets *AXL*, regulates the AKT/GSK-3β/β-catenin/Snail signaling cascade, and inhibits MMP-2/9	Oral	Li et al. (2018)
ANTI-TUMOR				

## Conclusion and Perspectives

Exosomes loaded with miRNAs and lncRNAs are a key communication pathway between CAFs and the different elements of the TME. Its relevance lies mainly in the promoter effect of protumoral and antitumoral events that modulate the genesis and the progression of various neoplasms. In the present work, the conventional effect of two types of exosomal ncRNAs derived from CAFs: miRNAs and lncRNAs, has been reviewed; this effect inhibits the translation by binding to its target mRNAs (Dragomir et al., 2018). Considering that cancer cells need different stimuli to form tumors and that CAFs, through the secretion of exosomal ncRNAs, among other mechanisms, can contribute significantly by favoring protumoral events, this type of extracellular vesicles derived from CAFs loaded with miRNAs and lncRNAs can be considered as a potential therapeutic target to prevent the development of cancer. Nevertheless, despite the increase in the last 5 years of strong evidence suggesting CAFs-derived exosomal ncRNAs as therapeutic targets, more studies are needed to confirm the protumoral or antitumoral effects exerted by the various miRNAs and lncRNAs. Probably like miRNAs, lncRNAs have unconventional effects on gene expression that are still awaiting to be studied.

Regardless of future studies, the translational way from the laboratory to the clinic has begun. At present, an extensive database of ncRNAs in extracellular vesicles called EVAtlas, a product of enormous work, is available (Liu et al., 2021). In the site http://bioinfo.life.hust.edu.cn/EVAtlas, different characteristics such as extracellular vesicle types and isolation methods, expression level, functions, related drugs, and target genes of seven types of ncRNA of human extracellular vesicles can be consulted, as well as the pathological or physiological condition, tissues, cells, and biological fluids from which they have been isolated. This great bioinformatic tool could be part of the foundations to transfer the findings found *in vitro*, *ex vivo* and, *in silico* on the modulating effects of exosomal ncRNAs, derived and not derived from CAFs in the development of neoplasms, towards therapeutic regimens that improve the prognosis of cancer patients.

Although it is true that it will not be an easy task to incorporate miRNAs and lncRNAs as targets or tools in cancer treatment schemes, their relevance should not be underestimated. In the analysis carried out by GLOBOCAN (Ferlay et al., 2019; Ferlay et al., 2020), the global incidence and mortality rates of different cancers show an increase as the years go by, highlighting the need for additional options than those currently available to help in the fight against neoplasms. Designing and performing safe clinical trials, in which miRNAs and lncRNAs are used as therapeutic tools, is a great challenge and it will be essential to consider them a new real therapeutic alternative that benefits oncological patients.

## References

[B1] Achreja A. Zhao H. Yang L. Yun T. H. Marini J. Nagrath D. (2017). Exo-MFA - A 13C Metabolic Flux Analysis Framework to Dissect Tumor Microenvironment-Secreted Exosome Contributions Towards Cancer Cell Metabolism. Metab. Eng. 43 (Pt B), 156–172. 10.1016/j.ymben.2017.01.001 28087332 PMC7393794

[B2] Admyre C. Johansson S. M. Qazi K. R. Filén J.-J. Lahesmaa R. Norman M. (2007). Exosomes With Immune Modulatory Features Are Present in Human Breast Milk. J. Immunol. 179 (3), 1969–1978. 10.4049/jimmunol.179.3.1969 17641064

[B3] Ali S. Suresh R. Banerjee S. Bao B. Xu Z. Wilson J. (2015). Contribution of microRNAs in Understanding the Pancreatic Tumor Microenvironment Involving Cancer Associated Stellate and Fibroblast Cells. Am. J. Cancer Res. 5 (3), 1251–1264. 26046003 PMC4449452

[B4] Amit M. Takahashi H. Dragomir M. P. Lindemann A. Gleber-Netto F. O. Pickering C. R. (2020). Loss of P53 Drives Neuron Reprogramming in Head and Neck Cancer. Nature. 578 (7795), 449–454. 10.1038/s41586-020-1996-3 32051587 PMC9723538

[B5] Baroni S. Romero-Cordoba S. Plantamura I. Dugo M. D’Ippolito E. Cataldo A. (2016). Exosome-Mediated Delivery of miR-9 Induces Cancer-Associated Fibroblast-Like Properties in Human Breast Fibroblasts. Cell Death Dis. 7 (7), e2312. 10.1038/cddis.2016.224 27468688 PMC4973361

[B6] Beermann J. Piccoli M.-T. Viereck J. Thum T. (2016). Non-Coding RNAs in Development and Disease: Background, Mechanisms, and Therapeutic Approaches. Physiol. Rev. 96 (4), 1297–1325. 10.1152/physrev.00041.2015 27535639

[B7] Chen B. Sang Y. Song X. Zhang D. Wang L. Zhao W. (2021). Exosomal miR-500a-5p Derived From Cancer-Associated Fibroblasts Promotes Breast Cancer Cell Proliferation and Metastasis through Targeting USP28. Theranostics. 11 (8), 3932–3947. 10.7150/thno.53412 33664871 PMC7914354

[B8] Chen J.-H. Wu A. T. H. Bamodu O. A. Yadav V. K. Chao T.-Y. Tzeng Y.-M. (2019). Ovatodiolide Suppresses Oral Cancer Malignancy by Down-Regulating Exosomal Mir-21/stat3/β-Catenin Cargo and Preventing Oncogenic Transformation of Normal Gingival Fibroblasts. Cancers. 12, 56. 10.3390/cancers12010056 PMC7017298 31878245

[B9] Cheng Q. Li X. Liu J. Ye Q. Chen Y. Tan S. (2017). Multiple Myeloma-Derived Exosomes Regulate the Functions of Mesenchymal Stem Cells Partially via Modulating miR-21 and miR-146a. Stem Cell Int. 2017, 1–9. 10.1155/2017/9012152 PMC5733127 29333170

[B10] Dayan D. Salo T. Salo S. Nyberg P. Nurmenniemi S. Costea D. E. (2012). Molecular Crosstalk Between Cancer Cells and Tumor Microenvironment Components Suggests Potential Targets for New Therapeutic Approaches in Mobile Tongue Cancer. Cancer Med. 1 (2), 128–140. 10.1002/cam4.24 23342263 PMC3544451

[B11] De Wever O. Van Bockstal M. Mareel M. Hendrix A. Bracke M. (2014). Carcinoma-Associated Fibroblasts Provide Operational Flexibility in Metastasis. Semin. Cancer Biol. 25, 33–46. 10.1016/j.semcancer.2013.12.009 24406210

[B12] Ding L. Ren J. Zhang D. Li Y. Huang X. Hu Q. (2018). A Novel Stromal lncRNA Signature Reprograms Fibroblasts to Promote the Growth of Oral Squamous Cell Carcinoma via LncRNA-CAF/Interleukin-33. Carcinogenesis. 39 (3), 397–406. 10.1093/carcin/bgy006 29346528

[B13] Domvri K. Petanidis S. Anestakis D. Porpodis K. Bai C. Zarogoulidis P. (2020). Exosomal lncRNA PCAT-1 Promotes Kras-Associated Chemoresistance via Immunosuppressive miR-182/miR-217 Signaling and p27/CDK6 Regulation. Oncotarget. 11 (29), 2847–2862. 10.18632/oncotarget.27675 32754302 PMC7381096

[B14] Donnarumma E. Fiore D. Nappa M. Roscigno G. Adamo A. Iaboni M. (2017). Cancer-associated Fibroblasts Release Exosomal microRNAs That Dictate an Aggressive Phenotype in Breast Cancer. Oncotarget. 8 (12), 19592–19608. 10.18632/oncotarget.14752 28121625 PMC5386708

[B15] Dou D. Ren X. Han M. Xu X. Ge X. Gu Y. (2020). Cancer-Associated Fibroblasts-Derived Exosomes Suppress Immune Cell Function in Breast Cancer via the miR-92/pd-L1 Pathway. Front. Immunol. 11, 2026. 10.3389/fimmu.2020.02026 33162971 PMC7581790

[B16] Dragomir M. P. Knutsen E. Calin G. A. (2018). SnapShot: Unconventional miRNA Functions. Cell. 174 (4), 1038. 10.1016/j.cell.2018.07.040 30096304

[B17] Elashiry M. Elsayed R. Elashiry M. M. Rashid M. H. Ara R. Arbab A. S. (2021). Proteomic Characterization, Biodistribution, and Functional Studies of Immune-Therapeutic Exosomes: Implications for Inflammatory Lung Diseases. Front. Immunol. 12, 636222. 10.3389/fimmu.2021.636222 33841418 PMC8027247

[B18] Fang T. Lv H. Lv G. Li T. Wang C. Han Q. (2018). Tumor-Derived Exosomal miR-1247-3p Induces Cancer-Associated Fibroblast Activation to foster Lung Metastasis of Liver Cancer. Nat. Commun. 9 (1), 191. 10.1038/s41467-017-02583-0 29335551 PMC5768693

[B19] Ferlay J. Colombet M. Soerjomataram I. Mathers C. Parkin D. M. Piñeros M. (2019). Estimating the Global Cancer Incidence and Mortality in 2018: GLOBOCAN Sources and Methods. Int. J. Cancer. 144 (8), 1941–1953. 10.1002/ijc.31937 30350310

[B20] Ferlay J. Laversanne M. Ervik M. Lam F. Colombet M. Mery L. (2020). Global Cancer Observatory: Cancer Tomorrow. Lyon, France: International Agency for Research on Cancer. Available at: https://gco.iarc.fr/tomorrow (Accessed 20 May, 2021).

[B21] Fiori M. E. Di Franco S. Villanova L. Bianca P. Stassi G. De Maria R. (2019). Cancer-Associated Fibroblasts as Abettors of Tumor Progression at the Crossroads of EMT and Therapy Resistance. Mol. Cancer. 18, 70. 10.1186/s12943-019-0994-2 30927908 PMC6441236

[B22] Gao Q. Fang X. Chen Y. Li Z. Wang M. (2021). Exosomal lncRNA UCA1 From Cancer-Associated Fibroblasts Enhances Chemoresistance in Vulvar Squamous Cell Carcinoma Cells. J. Obstet. Gynaecol. Res. 47 (1), 73–87. 10.1111/jog.14418 32812305

[B23] Gao Y. Li X. Zeng C. Liu C. Hao Q. Li W. (2020). CD63 + Cancer-Associated Fibroblasts Confer Tamoxifen Resistance to Breast Cancer Cells Through Exosomal miR-22. Adv. Sci. 7 (21), 2002518. 10.1002/advs.202002518 PMC7610308 33173749

[B24] Gener Lahav T. Adler O. Zait Y. Shani O. Amer M. Doron H. (2019). Melanoma-Derived Extracellular Vesicles Instigate Proinflammatory Signaling in the Metastatic Microenvironment. Int. J. Cancer. 145 (9), 2521–2534. 10.1002/ijc.32521 31216364

[B25] Goulet C. R. Champagne A. Bernard G. Vandal D. Chabaud S. Pouliot F. (2019). Cancer-Associated Fibroblasts Induce Epithelial-Mesenchymal Transition of Bladder Cancer Cells through Paracrine IL-6 Signalling. BMC Cancer. 19, 137. 10.1186/s12885-019-5353-6 30744595 PMC6371428

[B26] Gu J. Qian H. Shen L. Zhang X. Zhu W. Huang L. (2012). Gastric Cancer Exosomes Trigger Differentiation of Umbilical Cord Derived Mesenchymal Stem Cells to Carcinoma-Associated Fibroblasts Through TGF-β/Smad Pathway. PLoS One. 7 (12), e52465. 10.1371/journal.pone.0052465 23285052 PMC3527492

[B27] Guo H. Ha C. Dong H. Yang Z. Ma Y. Ding Y. (2019). Cancer-Associated Fibroblast-Derived Exosomal MicroRNA-98-5p Promotes Cisplatin Resistance in Ovarian Cancer by Targeting CDKN1A. Cancer Cel Int. 19, 347. 10.1186/s12935-019-1051-3 PMC6925473 31889899

[B28] Han K.-Y. Tran J. A. Chang J.-H. Azar D. T. Zieske J. D. (2017). Potential Role of Corneal Epithelial Cell-Derived Exosomes in Corneal Wound Healing and Neovascularization. Sci. Rep. 7, 40548. 10.1038/srep40548 28165027 PMC5292698

[B29] Herrera M. Llorens C. Rodríguez M. Herrera A. Ramos R. Gil B. (2018). Differential Distribution and Enrichment of Non-Coding RNAs in Exosomes From Normal and Cancer-Associated Fibroblasts in Colorectal Cancer. Mol. Cancer. 17, 114. 10.1186/s12943-018-0863-4 30075793 PMC6091058

[B30] Hu T. Hu J. (2019). Melanoma-derived Exosomes Induce Reprogramming Fibroblasts Into Cancer-Associated Fibroblasts via Gm26809 Delivery. Cell Cycle. 18 (22), 3085–3094. 10.1080/15384101.2019.1669380 31544590 PMC6816427

[B31] Huang T. X. Guan X. Y. Fu L. (2019). Therapeutic Targeting of the Crosstalk Between Cancer-Associated Fibroblasts and Cancer Stem Cells. Am. J. Cancer Res. 9 (9), 1889–1904. 31598393 PMC6780671

[B32] Huang Y. J. Huang T. H. Yadav V. K. Sumitra M. R. Tzeng D. T. Wei P. L. (2020). Preclinical Investigation of Ovatodiolide as a Potential Inhibitor of colon Cancer Stem Cells via Downregulating Sphere-Derived Exosomal β-Catenin/STAT3/miR-1246 Cargoes. Am. J. Cancer Res. 1010 (128), 46402337–46422354.

[B33] Jung W.-H. Yam N. Chen C.-C. Elawad K. Hu B. Chen Y. (2020). Force-Dependent Extracellular Matrix Remodeling by Early-Stage Cancer Cells Alters Diffusion and Induces Carcinoma-Associated Fibroblasts. Biomaterials. 234, 119756. 10.1016/j.biomaterials.2020.119756 31954229

[B34] Kim J. E. Kim B. G. Jang Y. Kang S. Lee J. H. Cho N. H. (2020). The Stromal Loss of miR-4516 Promotes the FOSL1-dependent Proliferation and Malignancy of Triple Negative Breast Cancer. Cancer Lett. 469, 256–265. 10.1016/j.canlet.2019.10.039 31672492

[B35] Lee J.-C. Wu A. T. H. Chen J.-H. Huang W.-Y. Lawal B. Mokgautsi N. (2020). HNC0014, a Multi-Targeted Small-Molecule, Inhibits Head and Neck Squamous Cell Carcinoma by Suppressing C-Met/STAT3/CD44/PD-L1 Oncoimmune Signature and Eliciting Antitumor Immune Responses. Cancers. 12, 3759. 10.3390/cancers12123759 PMC7764918 33327484

[B36] Li B. L. Lu W. Qu J. J. Ye L. Du G. Q. Wan X. P. (2019). Loss of Exosomal miR-148b From Cancer-Associated Fibroblasts Promotes Endometrial Cancer Cell Invasion and Cancer Metastasis. J. Cel Physiol. 234 (3), 2943–2953. 10.1002/jcp.27111 30146796

[B37] Li K. Liu T. Chen J. Ni H. Li W. (2020a). Survivin in Breast Cancer-Derived Exosomes Activates Fibroblasts by Up-Regulating SOD1, Whose Feedback Promotes Cancer Proliferation and Metastasis. J. Biol. Chem. 295 (40), 13737–13752. 10.1074/jbc.RA120.013805 32709750 PMC7535909

[B38] Li Y. Zhao Z. Liu W. Li X. (2020b). SNHG3 Functions as miRNA Sponge to Promote Breast Cancer Cells Growth Through the Metabolic Reprogramming. Appl. Biochem. Biotechnol. 191 (3), 1084–1099. 10.1007/s12010-020-03244-7 31956955 PMC7320061

[B39] Li W. Zhang X. Wang J. Li M. Cao C. Tan J. (2017). TGFβ1 in Fibroblasts-Derived Exosomes Promotes Epithelial-Mesenchymal Transition of Ovarian Cancer Cells. Oncotarget. 8 (56), 96035–96047. 10.18632/oncotarget.21635 29221185 PMC5707079

[B40] Li Y.-y. Tao Y.-w. Gao S. Li P. Zheng J.-m. Zhang S.-e. (2018). Cancer-Associated Fibroblasts Contribute to Oral Cancer Cells Proliferation and Metastasis via Exosome-Mediated Paracrine miR-34a-5p. EBioMedicine. 36, 209–220. 10.1016/j.ebiom.2018.09.006 30243489 PMC6197737

[B41] Liu C.-J. Xie G.-Y. Miao Y.-R. Xia M. Wang Y. Lei Q. (2021). EVAtlas: a Comprehensive Database for ncRNA Expression in Human Extracellular Vesicles. Nucleic Acids Res., 1–7. 10.1093/nar/gkab668 34387689 PMC8728297

[B42] Liu L. Zhang Z. Zhou L. Hu L. Yin C. Qing D. (2020). Cancer Associated Fibroblasts-Derived Exosomes Contribute to Radioresistance Through Promoting Colorectal Cancer Stem Cells Phenotype. Exp. Cel Res. 391 (2), 111956. 10.1016/j.yexcr.2020.111956 32169425

[B43] Luga V. Wrana J. L. (2013). Tumor-Stroma Interaction: Revealing Fibroblast-Secreted Exosomes as Potent Regulators of Wnt-Planar Cell Polarity Signaling in Cancer Metastasis. Cancer Res. 73 (23), 6843–6847. 10.1158/0008-5472.CAN-13-1791 24265274

[B44] Lv B. Zhu W. Feng C. (2020). Coptisine Blocks Secretion of Exosomal Circcct3 From Cancer-Associated Fibroblasts to Reprogram Glucose Metabolism in Hepatocellular Carcinoma. DNA Cel Biol. 39 (12), 2281–2288. 10.1089/dna.2020.6058 33001706

[B45] Mashouri L. Yousefi H. Aref A. R. Ahadi A. m. Molaei F. Alahari S. K. (2019). Exosomes: Composition, Biogenesis, and Mechanisms in Cancer Metastasis and Drug Resistance. Mol. Cancer. 18, 75. 10.1186/s12943-019-0991-5 30940145 PMC6444571

[B46] Masoud G. N. Li W. (2015). HIF-1α Pathway: Role, Regulation and Intervention for Cancer Therapy. Acta Pharmaceutica Sinica B. 5 (5), 378–389. 10.1016/j.apsb.2015.05.007 26579469 PMC4629436

[B47] Mathivanan S. Ji H. Simpson R. J. (2010). Exosomes: Extracellular Organelles Important in Intercellular Communication. J. Proteomics. 73 (10), 1907–1920. 10.1016/j.jprot.2010.06.006 20601276

[B48] Nilsson J. Skog J. Nordstrand A. Baranov V. Mincheva-Nilsson L. Breakefield X. O. (2009). Prostate Cancer-Derived Urine Exosomes: a Novel Approach to Biomarkers for Prostate Cancer. Br. J. Cancer. 100 (10), 1603–1607. 10.1038/sj.bjc.6605058 19401683 PMC2696767

[B49] Nouraee N. Khazaei S. Vasei M. Razavipour S. F. Sadeghizadeh M. Mowla S. J. (2016). MicroRNAs Contribution in Tumor Microenvironment of Esophageal Cancer. Cbm. 16 (3), 367–376. 10.3233/CBM-160575 PMC13016487 26889983

[B50] Pathania R. Ramachandran S. Elangovan S. Padia R. Yang P. Cinghu S. (2015). DNMT1 Is Essential for Mammary and Cancer Stem Cell Maintenance and Tumorigenesis. Nat. Commun. 6, 6910. 10.1038/ncomms7910 25908435 PMC4410389

[B51] Principe S. Mejia-Guerrero S. Ignatchenko V. Sinha A. Ignatchenko A. Shi W. (2018). Proteomic Analysis of Cancer-Associated Fibroblasts Reveals a Paracrine Role for MFAP5 in Human Oral Tongue Squamous Cell Carcinoma. J. Proteome Res. 17 (6), 2045–2059. 10.1021/acs.jproteome.7b00925 29681158

[B52] Qin X. Guo H. Wang X. Zhu X. Yan M. Wang X. (2019). Exosomal miR-196a Derived from Cancer-Associated Fibroblasts Confers Cisplatin Resistance in Head and Neck Cancer Through Targeting CDKN1B and ING5. Genome Biol. 20, 12. 10.1186/s13059-018-1604-0 30642385 PMC6332863

[B53] Ramteke A. Ting H. Agarwal C. Mateen S. Somasagara R. Hussain A. (2015). Exosomes Secreted under Hypoxia Enhance Invasiveness and Stemness of Prostate Cancer Cells by Targeting Adherens junction Molecules. Mol. Carcinog. 54 (7), 554–565. 10.1002/mc.22124 24347249 PMC4706761

[B54] Ren J. Ding L. Zhang D. Shi G. Xu Q. Shen S. (2018). Carcinoma-Associated Fibroblasts Promote the Stemness and Chemoresistance of Colorectal Cancer by Transferring Exosomal lncRNA H19. Theranostics. 8 (14), 3932–3948. 10.7150/thno.25541 30083271 PMC6071523

[B55] Richards K. E. Zeleniak A. E. Fishel M. L. Wu J. Littlepage L. E. Hill R. (2017). Cancer-Associated Fibroblast Exosomes Regulate Survival and Proliferation of Pancreatic Cancer Cells. Oncogene. 36 (13), 1770–1778. 10.1038/onc.2016.353 27669441 PMC5366272

[B56] Ringuette Goulet C. Bernard G. Tremblay S. Chabaud S. Bolduc S. Pouliot F. (2018). Exosomes Induce Fibroblast Differentiation Into Cancer-Associated Fibroblasts Through TGFβ Signaling. Mol. Cancer Res. 16 (7), 1196–1204. 10.1158/1541-7786.MCR-17-0784 29636362

[B57] Sansone P. Savini C. Kurelac I. Chang Q. Amato L. B. Strillacci A. (2017). Packaging and Transfer of Mitochondrial DNA via Exosomes Regulate Escape From Dormancy in Hormonal Therapy-Resistant Breast Cancer. Proc. Natl. Acad. Sci. USA. 114 (43), E9066–E9075. 10.1073/pnas.1704862114 29073103 PMC5664494

[B58] Seo N. Akiyoshi K. Shiku H. (2018). Exosome-Mediated Regulation of Tumor Immunology. Cancer Sci. 109 (10), 2998–3004. 10.1111/cas.13735 29999574 PMC6172045

[B59] Shah S. H. Miller P. Garcia-Contreras M. Ao Z. Machlin L. Issa E. (2015). Hierarchical Paracrine Interaction of Breast Cancer Associated Fibroblasts With Cancer Cells via hMAPK-microRNAs to Drive ER-Negative Breast Cancer Phenotype. Cancer Biol. Ther. 16 (11), 1671–1681. 10.1080/15384047.2015.1071742 26186233 PMC4846097

[B60] Shan G. Gu J. Zhou D. Li L. Cheng W. Wang Y. (2020). Cancer-Associated Fibroblast-Secreted Exosomal miR-423-5p Promotes Chemotherapy Resistance in Prostate Cancer by Targeting GREM2 Through the TGF-β Signaling Pathway. Exp. Mol. Med. 52 (11), 1809–1822. 10.1038/s12276-020-0431-z 33144675 PMC8080786

[B61] Shan G. Zhou X. Gu J. Zhou D. Cheng W. Wu H. (2021). Downregulated Exosomal microRNA-148b-3p in Cancer Associated Fibroblasts Enhance Chemosensitivity of Bladder Cancer Cells by Downregulating the Wnt/β-Catenin Pathway and Upregulating PTEN. Cell Oncol. 44, 45–59. 10.1007/s13402-020-00500-0 PMC7906940 33423167

[B62] Shimoda M. Principe S. Jackson H. W. Luga V. Fang H. Molyneux S. D. (2014). Loss of the Timp Gene Family Is Sufficient for the Acquisition of the CAF-Like Cell State. Nat. Cel Biol. 16 (9), 889–901. 10.1038/ncb3021 25150980

[B63] Sui C. Meng F. Li Y. Jiang Y. (2015). miR-148b Reverses Cisplatin-Resistance in Non-Small Cell Cancer Cells via Negatively Regulating DNA (Cytosine-5)-Methyltransferase 1(DNMT1) Expression. J. Transl. Med. 13, 132. 10.1186/s12967-015-0488-y 25927928 PMC4417300

[B64] Sun L. P. Xu K. Cui J. Yuan D. Y. Zou B. Li J. (2019). Cancer associated Fibroblast derived Exosomal miR3825p Promotes the Migration and Invasion of Oral Squamous Cell Carcinoma. Oncol. Rep. 42 (4), 1319–1328. 10.3892/or.2019.7255 31364748 PMC6718099

[B65] Sun W. Fu S. (2019). Role of Cancer-Associated Fibroblasts in Tumor Structure, Composition and the Microenvironment in Ovarian Cancer (Review). Oncol. Lett. 18 (3), 2173–2178. 10.3892/ol.2019.10587 31452720 PMC6676664

[B66] Théry C. (2011). Exosomes: Secreted Vesicles and Intercellular Communications. F1000 Biol. Rep. 3, 15. 10.3410/B3-15 21876726 PMC3155154

[B67] Vered M. Lehtonen M. Hotakainen L. Pirilä E. Teppo S. Nyberg P. (2015). Caveolin-1 Accumulation in the Tongue Cancer Tumor Microenvironment Is Significantly Associated with Poor Prognosis: an *In-Vivo* and *In-Vitro* Study. BMC Cancer. 15, 25. 10.1186/s12885-015-1030-6 25633184 PMC4318139

[B68] Wang H. Wei H. Wang J. Li L. Chen A. Li Z. (2020). MicroRNA-181d-5p-Containing Exosomes Derived From CAFs Promote EMT by Regulating CDX2/HOXA5 in Breast Cancer. Mol. Ther. - Nucleic Acids. 19, 654–667. 10.1016/j.omtn.2019.11.024 31955007 PMC6970169

[B69] Wang J.-W. Wu X.-F. Gu X.-J. Jiang X.-H. (2019a). Exosomal miR-1228 From Cancer-Associated Fibroblasts Promotes Cell Migration and Invasion of Osteosarcoma by Directly Targeting SCAI. Oncol. Res. 27 (9), 979–986. 10.3727/096504018X15336368805108 30180920 PMC7848259

[B70] Wang X. Qin X. Yan M. Shi J. Xu Q. Li Z. (2019b). Loss of Exosomal miR-3188 in Cancer-Associated Fibroblasts Contributes to HNC Progression. J. Exp. Clin. Cancer Res. 38, 151. 10.1186/s13046-019-1144-9 30961650 PMC6454737

[B71] Wu H.-J. Hao M. Yeo S. K. Guan J.-L. (2020). FAK Signaling in Cancer-Associated Fibroblasts Promotes Breast Cancer Cell Migration and Metastasis by Exosomal miRNAs-Mediated Intercellular Communication. Oncogene. 39 (12), 2539–2549. 10.1038/s41388-020-1162-2 31988451 PMC7310603

[B72] Yang Y. Li J. Geng Y. (2020). Exosomes Derived from Chronic Lymphocytic Leukaemia Cells Transfer miR-146a to Induce the Transition of Mesenchymal Stromal Cells into Cancer-Associated Fibroblasts. J. Biochem. 168 (5), 491–498. 10.1093/jb/mvaa064 32770182

[B73] Yeon J. H. Jeong H. E. Seo H. Cho S. Kim K. Na D. (2018). Cancer-Derived Exosomes Trigger Endothelial to Mesenchymal Transition Followed by the Induction of Cancer-Associated Fibroblasts. Acta Biomater. 76, 146–153. 10.1016/j.actbio.2018.07.001 30078422

[B74] You J. Li M. Cao L. M. Gu Q. H. Deng P. B. Tan Y. (2019). Snail1-Dependent Cancer-Associated Fibroblasts Induce Epithelial-Mesenchymal Transition in Lung Cancer Cells via Exosomes. QJM. 112 (8), 581–590. 10.1093/qjmed/hcz093 31106370

[B75] Zhang H. Deng T. Liu R. Ning T. Yang H. Liu D. (2020a). CAF Secreted miR-522 Suppresses Ferroptosis and Promotes Acquired Chemo-Resistance in Gastric Cancer. Mol. Cancer. 19, 43. 10.1186/s12943-020-01168-8 32106859 PMC7045485

[B76] Zhang N. Wang Y. Liu H. Shen W. (2020b). Extracellular Vesicle Encapsulated microRNA-320a Inhibits Endometrial Cancer by Suppression of the HIF1α/VEGFA axis. Exp. Cel Res. 394 (2), 112113. 10.1016/j.yexcr.2020.112113 32473223

[B77] Zhang H. W. Shi Y. Liu J. B. Wang H. M. Wang P. Y. Wu Z. J. (2021). Cancer-associated Fibroblast-Derived Exosomal microRNA-24-3p Enhances Colon Cancer Cell Resistance to MTX by Down-Regulating CDX2/HEPH axis. J. Cel Mol. Med. 25 (8), 3699–3713. 10.1111/jcmm.15765 PMC8051723 33621425

[B78] Zhang Y.-F. Zhou Y.-Z. Zhang B. Huang S.-F. Li P.-P. He X.-M. (2019a). Pancreatic Cancer-Derived Exosomes Promoted Pancreatic Stellate Cells Recruitment by Pancreatic Cancer. J. Cancer. 10 (18), 4397–4407. 10.7150/jca.27590 31413760 PMC6691697

[B79] Zhang Y. Cai H. Chen S. Sun D. Zhang D. He Y. (2019b). Exosomal Transfer of miR-124 Inhibits normal Fibroblasts to Cancer-Associated Fibroblasts Transition by Targeting Sphingosine Kinase 1 in Ovarian Cancer. J. Cel Biochem. 120 (8), 13187–13201. 10.1002/jcb.28593 30957275

[B80] Zhang Z. Li X. Sun W. Yue S. Yang J. Li J. (2017). Loss of Exosomal miR-320a From Cancer-Associated Fibroblasts Contributes to HCC Proliferation and Metastasis. Cancer Lett. 397, 33–42. 10.1016/j.canlet.2017.03.004 28288874

[B81] Zhao G. Li H. Guo Q. Zhou A. Wang X. Li P. (2020). Exosomal Sonic Hedgehog Derived From Cancer-Associated Fibroblasts Promotes Proliferation and Migration of Esophageal Squamous Cell Carcinoma. Cancer Med. 9 (7), 2500–2513. 10.1002/cam4.2873 32030915 PMC7131837

[B82] Zhao H. Yang L. Baddour J. Achreja A. Bernard V. Moss T. (2016). Tumor Microenvironment Derived Exosomes Pleiotropically Modulate Cancer Cell Metabolism. Elife. 5, e10250. 10.7554/eLife.10250 26920219 PMC4841778

[B83] Zhao Q. Huang L. Qin G. Qiao Y. Ren F. Shen C. (2021). Cancer-associated Fibroblasts Induce Monocytic Myeloid-Derived Suppressor Cell Generation via IL-6/Exosomal miR-21-Activated STAT3 Signaling to Promote Cisplatin Resistance in Esophageal Squamous Cell Carcinoma. Cancer Lett. 518, 35–48. 10.1016/j.canlet.2021.06.009 34139285

[B84] Zhou L. Li J. Tang Y. Yang M. (2021). Exosomal LncRNA LINC00659 Transferred From Cancer-Associated Fibroblasts Promotes Colorectal Cancer Cell Progression via miR-342-3p/ANXA2 axis. J. Transl. Med. 19, 8. 10.1186/s12967-020-02648-7 33407563 PMC7789760

